# Asymmetry in Mechanosensitive Gene Expression during Aortic Arch Morphogenesis

**DOI:** 10.1038/s41598-018-35127-7

**Published:** 2018-11-16

**Authors:** Cansu Karakaya, Selda Goktas, Merve Celik, William J. Kowalski, Bradley B. Keller, Kerem Pekkan

**Affiliations:** 1 Mechanical Engineering Department, Koç University, Istanbul, Turkey; 2 Kosair Charities Pediatric Heart Research Program, Cardiovascular Innovation Institute, University of Louisville, Louisville, KY United States of America

## Abstract

Embryonic aortic arches (AA) are initially bilaterally paired, transitional vessels and failures in remodeling based on hemodynamic and growth-related adaptations cause a spectrum of congenital heart disease (CHD) anatomies. Identifying regulatory mechanisms and cross-talk between the genetic elements of these vessels are critical to understand the ethiology of CHD and refine predictive computational models. This study aims to screen expression profiles of fundamental biological pathways in AA at early stages of chick embryo morphogenesis and correlate them with our current understanding of growth and mechanical loading. Reverse transcription-quantitative PCR (RT-qPCR) was followed by correlation and novel peak expression analyses to compare the behaviour and activation period of the genes. Available protein networks were also integrated to investigate the interactions between molecules and highlight major hierarchies. Only wall shear stress (WSS) and growth-correlated expression patterns were investigated. Effect of WSS was seen directly on angiogenesis as well on structural and apoptosis-related genes. Our time-resolved network suggested that WSS-correlated genes coordinate the activity of critical growth factors. Moreover, differential gene expression of left and right AA might be an indicator of subsequent asymmetric morphogenesis. These findings may further our understanding of the complex processes of cardiac morphogenesis and errors resulting in CHD.

## Introduction

Congenital heart disease (CHD) is the most common type of cardiac disorder and occurs in approximately 8 of every 1000 live births^1^. In order to survive, severely cyanotic patients require urgent open-heart surgery shortly after birth and further interventions throughout their lifetime^2^. CHDs develop due to either novel genetic mutations or epigenetic insults that alter cardiac morphogenesis *in utero*. Previous data have shown that embryonic cardiovascular systems dynamically regulate structure and function over very short time periods, and biomechanical loading conditions within the heart and aortic arches affect vascular morphology and the future genetic expression^3^.

Embryonic aortic arches (AA) are central in the prognosis of major CHD anatomies. AA vessels are transient and undergo regression and remodeling to form the great arteries of adult circulation^4^. At the embryonic stage, blood is transported from the heart to the dorsal aorta through the bilaterally paired AA system. Six paired AA appear symmetrically and consecutively, then they selectively regress and remodel during development resulting in an *asymmetric* vasculature during a relatively short time period. The cause of asymmetry in AA has been associated with the cardiac neural crest cells (NCC) which migrate, surround the AA and differentiate to secrete extracellular matrix (ECM)^5,6^. Moreover, asymmetric remodeling was related to selective apoptosis^7^ and paired-like homeodomain 2 (Pitx2) -mediated pathways^8^ which induce changes in blood flow through AA^9^, but more comprehensive investigations are needed.

In the normal development of humans, left AA IV persists, and eventually forms a segment of the mature aortic arch. Whereas, the right AA IV forms the proximal right subclavian artery. Moreover, the distal portion of left AA VI forms ductus arteriosus while right AA VI regresses. AA V exist as a portion of AA VI and do not contribute to mature system^10^. Other AA pairs give rise to more symmetrical patterns; AA I and II form portions of maxillary and stapedial arteries, respectively, AA III form the carotid arteries and proximal parts of AA VI form proximal segments of the pulmonary arteries. Aortic arch anomalies are hypothesized to correlate with the symmetry breaking patterns in AA regression and remodeling. For instance, the persistence of both left and right AA IV cause double aortic arch formation. Abnormal involution of left AA IV with the persistence of left AA VI result in right aortic arch with aberrant left subclavian artery. Abnormal regression of the right dorsal aorta causes the formation of left aortic arch with aberrant right subclavian artery. Infants with AA anomalies generally require surgical treatment for correction^4^. To understand the mechanism of these defects, chick embryos are widely used as model organisms since the AA development of chick embryos and humans is similar with a difference; right AA IV persists and forms the right-sided AA in chick embryos. Moreover, it is known that the genetic pathways controlling AA development in human and chick are comparable^11^.

**Figure 1:**
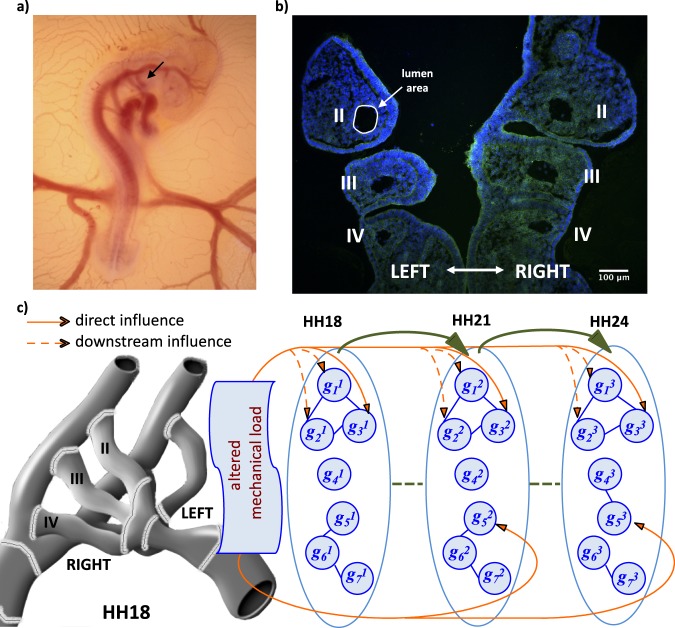
Overview of the study. (**a**) Lateral view of the chick embryo at stage HH18 is demonstrated. The arrow shows the AA vessels. (**b**) Immunofluorescence staining image of sectioned AA apparatus at HH18 is shown. Individual left and right AA are denoted with Roman numbers. The encircled area corresponds to lumen area of individual AA that are used for average diameter calculation. (**c**) Interactions of major molecular pathways and individual genes (*g*_*i*_) with the mechanical loading during three embryonic time-points are illustrated. Schematic shows the predicted effects of altered mechanical loading (only the effect of WSS was studied in this work) on different biological pathways at stages HH18, HH21 and HH24. AA apparatus is an example of the left and right individual AA observed at HH18.

In our previous descriptive studies, we measured the wall shear stress (WSS) and diameter of each AA in chick embryos at Hamburger-Hamilton stages (HH)^12^ 18 and 24^2^ and later HH21^13^. Three pairs of AA co-existed at HH18 and HH24; however, HH21 was a unique stage in which four different vascular configurations having two, three or four pairs of AA could be seen. All chick embryos included AA II, III, IV at HH18 and AA III, IV, VI at HH24. On the other hand, 2AA configuration of HH21 included AA III and IV; 4AA configuration included AA II, III, IV, VI; 3AA-cranial configuration was similar to HH18 and included AA II, III, IV; 3AA-caudal configuration was similar to HH24 and included AA III, IV, VI. In all cases, the AA system was symmetrical, so the left and right AA were observed together. On the other hand, there were significant changes in mechanical loading between stages and the diameter measures between left and right were significantly different at HH24 with dominance in right AA^2^. This asymmetric mechanical loading might, therefore, initiate the transition from symmetric to an asymmetric system, which would influence the mechanosensitive gene expression levels, further augmenting the asymmetric development (Fig. 1c).

Prior to further investigating this complex vascular apparatus, we studied the interactions between vascular growth, hemodynamics and gene expression in different developmental stages of chick embryo for the right vitelline artery (RVA)^14^. In this simpler arterial vessel, it was observed that the expression trends of angiogenesis- and apoptosis-related genes varied significantly despite the steady growth rates. WSS had a direct influence on vascular endothelial growth factor A (*VEGFA)* expression, but matrix metalloproteinase 2 (*MMP2)* and caspase 3 (*CASP3)* expressions were not affected by hemodynamic changes. In the same study, mechanosensitive genes krüppel-like factor 2 (*KLF2)* and nitric oxide synthase 3 (*NOS3)* were expressed proportionally to WSS whereas endothelin 1 (*ET1)* expression responded WSS with a significant time lag. Moreover, transforming growth factor beta 3 (*TGFβ3)* had an expression trend proportional to pulsatile pressure. The relations and different patterns in gene expression of RVA with time encouraged us to investigate and compare the gene expression patterns in the AA system.

There are a small number of studies on gene expression trends within the AA in relation to asymmetric development. Mechanosensitive gene expression was investigated in the cardiovascular system of chick embryos. As a general trend, *KLF2* and *NOS3* expressions were seen at high WSS areas while *ET1* was inversely related with WSS later in development. In AA, *KLF2* expression decreased, *ET1* expression increased and *NOS3* expression did not change between HH16-HH30^15^. In another study, it was shown that unilateral ablation of *Pitx2* expression damages asymmetric vascular development^9^. Moreover, *Pitx2* expression changes the morphology of the outflow tract which results in the altered blood flow and eventually changes in the expression of vascular endothelial and platelet-derived growth factor receptors.

In this study, we investigated the expression levels of genes related to angiogenesis, cardiovascular development and remodeling, ECM, cytoskeleton and apoptosis in AA of chick embryos at HH18, HH21 and HH24 to understand the correlation of gene expression with growth and hemodynamic changes and asymmetric development of AA system in later stages. Furthermore, a novel peak expression analysis of WSS-correlated genes allowed us to acquire integrated time-resolved information on the genetic pathways controlling AA development and morphogenesis, the first time in literature.

## Results

### WSS and Diameter

**Figure 2:**
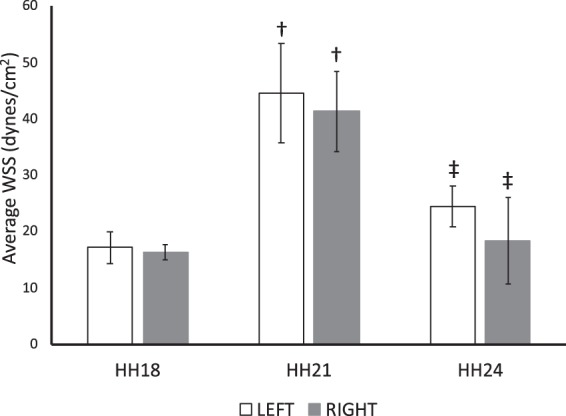
Calculated average WSS. Average WSS of left and right AA is shown for HH18, HH21 and HH24 with a peak at HH21. Following symbols indicate the statistically significant difference (*p* < *0*.*05*): ^†^for the difference in the same lateral between HH18 and HH21; ^‡^for the difference in the same lateral between HH21 and HH24.

**Figure 3:**
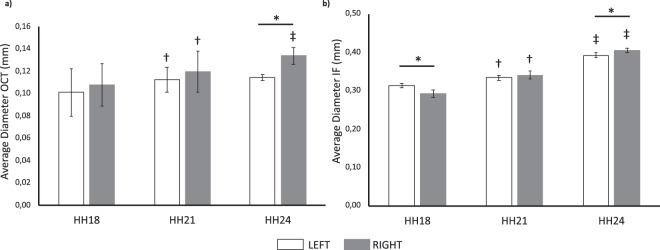
Calculated average diameters (**a**) Average diameters for left and right AA were calculated for HH18, HH21 and HH24 from our previous studies^2,13^. (**b**) Average diameters for left and right AA were calculated for HH18, HH21 and HH24 from immunofluorescence (IF) images. Following symbols indicate the statistically significant difference (*p* < *0*.*05*): ^†^for the difference in the same lateral between HH18 and HH21; ^‡^for the difference in the same lateral between HH21 and HH24; * for left and right AA differences within the same stage.

According to our calculations, which were further detailed in Methods section, the average-WSS increased from HH18 to HH24, reaching its peak at HH21 for both left and right AA (Fig. 2). WSS changes between HH18 and HH21 (p = 0.001 for left, p = 0.002 for right), and also between HH21 and HH24 (p = 0.013 for left, p = 0.005 for right) were statistically significant while there was not a significant change on different laterals within stages. Average diameter of AA at HH18, HH21 and HH24 were calculated from our previous optical coherence tomography (OCT) data and new immunofluorescence (IF) images (Fig. 3). Average diameter calculated from OCT data significantly increased from HH18 to HH21 (p = 0.007 for left, p = 0.019 for right) and increased further from HH21 and HH24 with a significant change for right AA (p < 0.001). Right AA diameter was significantly higher than left at HH24 (p < 0.001) (Fig. 3a). Average diameter calculated from IF images showed a similar significant increase through development (p < 0.001 for left and right AA between HH18-HH21 and HH21-HH24) (Fig. 3b). Moreover, significant differences were observed between laterals at HH18 (p < 0.001) and HH24 (p = 0.041).

### Gene expression

#### Angiogenesis genes

**Figure 4:**
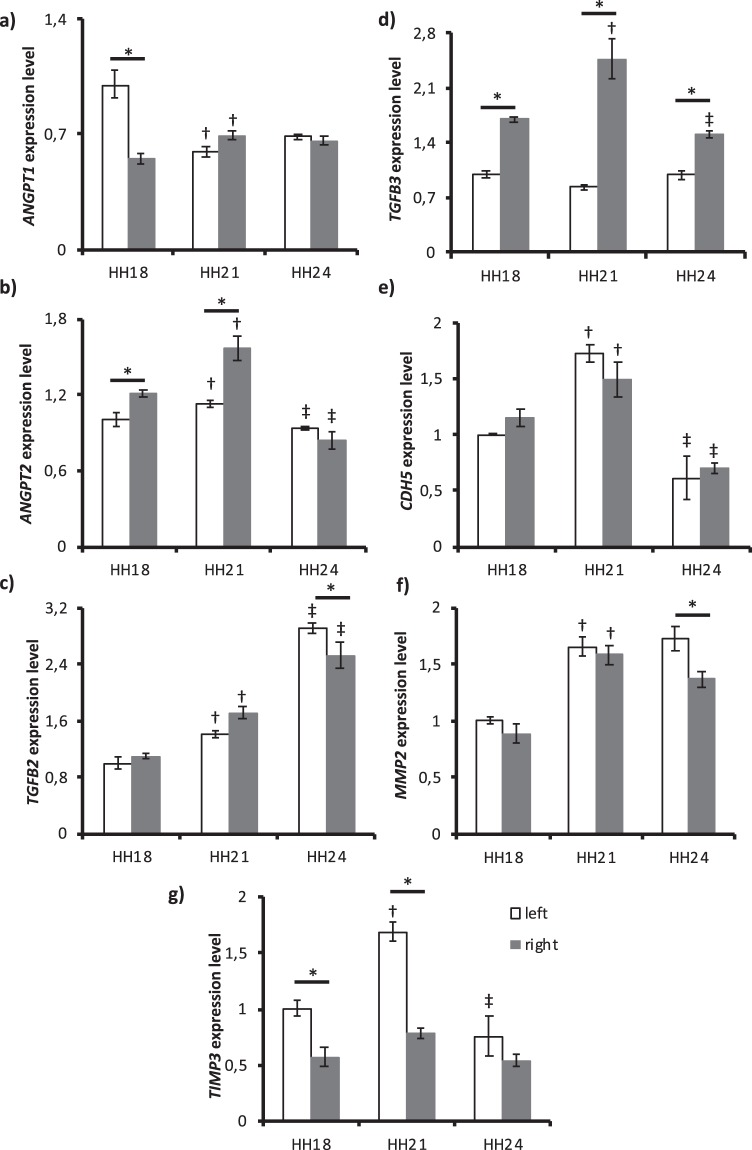
Expression levels of angiogenesis-related genes at HH18, HH21 and HH24. (**a**) *ANGPT1* (**b**) *ANGPT2* (**c**) *TGFβ2* (**d**) *TGFβ3* (**e**) *CDH5* (**f**) *MMP2* and (**g**) *TIMP3* expressions for left and right AA are shown. *GAPDH* gene is used for normalization and fold changes are determined based on HH18 left AA. Following symbols indicate the statistically significant difference (*p* < *0*.*05*): ^†^for the difference in the same lateral between HH18 and HH21; ^‡^for the difference in the same lateral between HH21 and HH24; * for left and right AA differences within the same stage.

Angiopoietin 1 *(ANGPT1)* expression (Fig. 4a) significantly decreased towards HH21 in left AA (p < 0.001) while expression increased in right AA (p = 0.017). Left AA expression was higher than the right AA expression at HH18 (p < 0.001). Angiopoietin 2 *(ANGPT2)* expression (Fig. 4b) significantly changed between all stages for right AA (p < 0.001 for both) and decreased towards HH24 for left AA (p = 0,007). Right AA expression was higher than left AA expression at HH18 (p = 0.005) and HH21 (p < 0.001). Transforming growth factor beta 2 (*TGFβ2)* (Fig. 4c) expression gradually increased over time for both left (p = 0.022 and p < 0.001) and right AA (p = 0.001 and p < 0.001) with a significant left-right difference at HH24 (p = 0.033). Although *TGFβ3* expression (Fig. 4d) remained constant for left AA, expression increased towards HH21 (p < 0.001) and decreased towards HH24 (p < 0.001) in right AA with very low p values within stages (p < 0.001 for all). Vascular-endothelial cadherin/cadherin 5 (*CDH5)* expression (Fig. 4e) significantly increased (p < 0.001 for left and p = 0.023 for right) and then decreased (p < 0.001 for left and right), while there was no change between left and right AA. *MMP2* expression (Fig. 4f) increased towards HH21 (p < 0.001 for left and right) and became higher in left AA at HH24 (p = 0.012). Tissue inhibitor of metalloproteinase 3 (*TIMP3)* expression (Fig. 4g) was significantly changed for left AA (p < 0.001 for both stages) and a considerable left-right difference was seen at HH18 (p = 0.001) and HH21 (p < 0.001).

#### Cardiovascular development and remodeling genes

**Figure 5:**
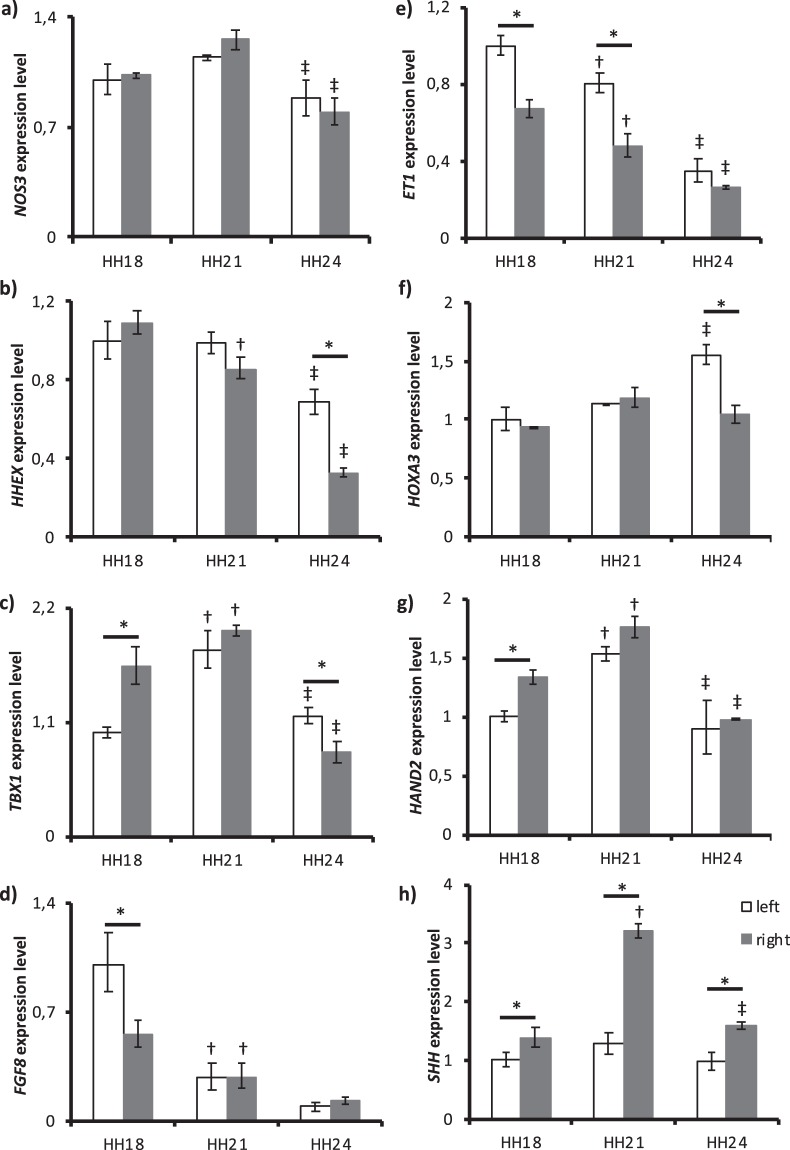
Expression levels of cardiovascular development and remodeling genes at HH18, HH21 and HH24. (**a**) *NOS3* (**b**) *HHEX* (**c**) *TBX1* (**d**) *FGF8* (**e**) *ET1* (**f**) *HOXA3* (**g**) *HAND2* and (**h**) *SHH* expressions for left and right AA are shown. *GAPDH* gene is used for normalization and fold changes are determined based on HH18 left AA. Following symbols indicate the statistically significant difference (*p* < *0*.*05*): ^†^for the difference in the same lateral between HH18 and HH21; ^‡^for the difference in the same lateral between HH21 and HH24; * for left and right AA differences within the same stage.

*NOS3* expression (Fig. 5a) significantly decreased from HH21 to HH24 (p = 0.026 for left and p < 0.001 for right) without any difference between left and right AA. Hematopoietically expressed homeobox (*HHEX)* expression (Fig. 5b) decreased over time with a significant difference between HH18 and HH21 for right AA (p = 0.005) and between HH21 and HH24 (p < 0.001 for left and right). Also, there was a significant change between left and right AA at HH24 (p < 0.001). T-box 1 (*TBX1)* expression (Fig. 5c) significantly increased from HH18 to HH21 (p < 0.001 for left and p = 0.009 right) and decreased from HH21 to HH24 (p < 0.001 for both left and right). Right AA expression was significantly higher at HH18 (p < 0.001) while left AA expression was dominant at HH24 (p = 0.007). Fibroblast growth factor 8 (*FGF8)* expression (Fig. 5d) in AA gradually decreased over time. The decrease was significant between HH18 and HH21 (p < 0.001 for left and p = 0.040 for right), and left AA expression was significantly higher at HH18 (p < 0.001). *ET1* expression (Fig. 5e) also decreased with time. A significant decrease was observed from HH18 to HH21 (p = 0.03 for left and p < 0.041 for right) and from HH21 to HH24 (p < 0.001 for left and p = 0.002 for right). Moreover, left AA expression was higher at HH18 and HH21 (p < 0.001 for both laterals). Homeobox A3 (*HOXA3)* expression (Fig. 5f) significantly increased only from HH21 to HH24 for left AA (p = 0.008). Furthermore, left AA expression was significantly higher than the right AA expression at HH24 (p = 0.002). For left AA, heart and neural crest derivates expressed 2 (*HAND2)* expression (Fig. 5g) increased from HH18 to HH21 with a following decrease from HH21 to HH24 (p < 0.001 for both). For right AA, *HAND2* expression increased from HH18 to HH21(p = 0.006) and decreased from HH21 to HH24 (p < 0.001). Also, the right AA expression was significantly higher at HH18 (p = 0.028). Sonic hedgehog (*SHH)* expression (Fig. 5h) was significant for the right AA with an increase from HH18 to HH21 followed by a decrease from HH21 to HH24 (p < 0.001 for both laterals). Right AA expression was higher for all stages (p = 0.041 at HH18, p < 0.001 at HH21 and p = 0.001 at HH24).

#### Extracellular Matrix, Cytoskeleton and Apoptosis genes

**Figure 6:**
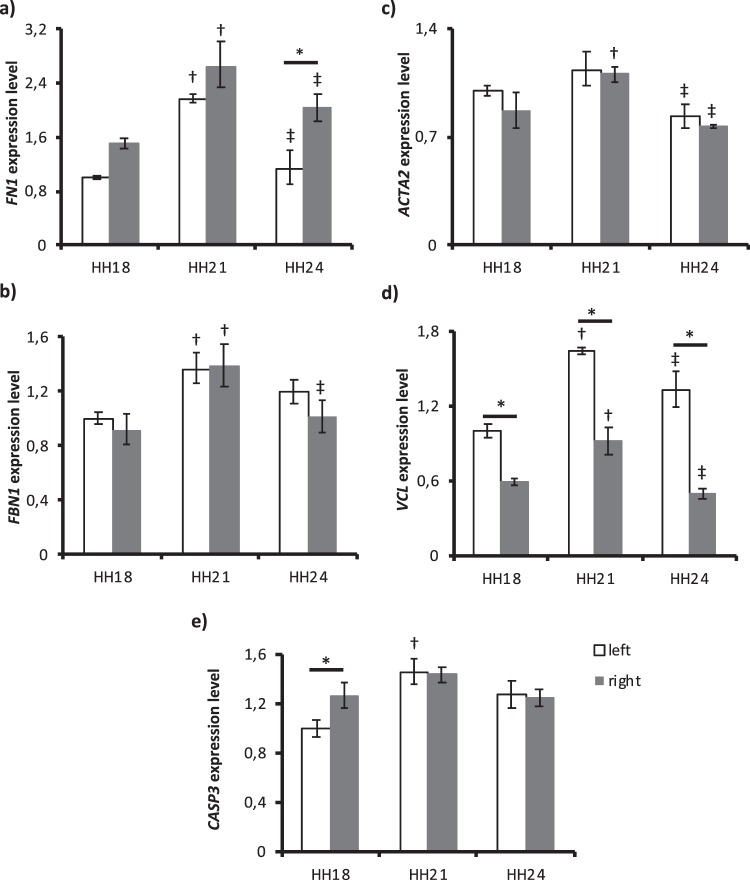
Expression levels of the extracellular matrix, cytoskeleton and apoptosis genes at HH18, HH21 and HH24. (**a**) *FN1* (**b**) *FBN1* (**c**) *ACTA2* (**d**) *VCL* and (**e**) *CASP3* expressions for left and right AA are shown. *GAPDH* gene is used for normalization and fold changes are determined based on HH18 left AA. Following symbols indicate the statistically significant difference (*p* < *0*.*05*): ^†^for the difference in the same lateral between HH18 and HH21; ^‡^for the difference in the same lateral between HH21 and HH24; * for left and right AA differences within the same stage.

Fibronectin 1 *(FN1)* expression (Fig. 6a) increased from HH18 to HH21 (p < 0.001 for both left and right) and decreased from HH21 to HH24 (p < 0.001 for left and p = 0.016 for right). Right AA expression was significantly higher at HH24 (p = 0.001). Fibrillin 1 (*FBN1)* expression (Fig. 6b) also significantly increased at first (p = 0.009 for left and p = 0.001 for right), and significantly decreased from HH21 to HH24 in right AA (p = 0.008). A significant increase in smooth muscle alpha actin (*ACTA2)* expression (Fig. 6c) from HH18 to HH21 was seen in right AA (p = 0.013), and a significant decrease from HH21 to HH24 was seen in both left (p = 0.002) and right AA (p = 0.001). Vinculin (*VCL)* expression (Fig. 6d) was significant at all stages. An increase in expression from HH18 to HH21 (p < 0.001 for left and p = 0.006 for right) was followed by a decrease from HH21 to HH24 (p = 0.007 for left and p < 0.001 for right). Expression in left AA was higher at all stages (p = 0.001 at HH18, p < 0.001 at HH21 and HH24). *CASP3* expression (Fig. 6e) significantly increased in left AA from HH18 to HH21 (p < 0.001). Also, the right AA expression was higher at HH18 (p = 0.016).

Expression results of collagen 1 *(COL1)*, *COL3*, *COL4*, tropoelastin (*ELN)* and *VEGFA* will be presented in another study; however, they were included into the following analyses in this study.

### Gene expression trends

Based on the average WSS and diameter calculations, two trends, WSS-trend and growth-trend, were determined. WSS-trend was the change in average WSS with an increase through HH21 following a decrease through HH24. Although the significant increase between HH21 and HH24 was not determined for left AA in average diameter calculation from OCT data, the significant difference was detected in average diameter calculated from IF images. Therefore, the continuous increase in average diameters calculated from OCT data was considered as growth-trend. Furthermore, gene expression patterns were classified based on their similarity to WSS or growth-trends. Considering the gene expression temporal cascade (Fig. 1c), the WSS-trend is upstream whereas the growth-trend is downstream.

### Correlation, peak expression and network development

Correlations for left and right AA were calculated based on the trends of the gene expressions. However, *HOXA3*, *MMP2* and *COL1* showed different trends on left and right AA, so they were grouped separately. Interestingly in this group of genes, left AA showed growth-trend and right AA showed WSS-trend, so they were correlated accordingly.

Most of the genes that we investigated were correlated strongly with averaged WSS. *FBN1*, *TGFβ3* and *SHH* demonstrated the highest average R-value of 0.97 among the WSS-correlated genes. *VCL*, *CASP3* and *FN1* followed them with an average R-value of 0.96. *ELN*, *COL4* and *TIMP3* correlations had average R values of 0.95, 0.94 and 0.92 whereas *HAND2* and *TBX1* had 0.88 and 0.84, respectively. Average R-value of 0.80 belongs to *ACTA2* and *CDH5* correlations while that of 0.78 corresponds to *NOS3* and *ANGPT1* correlations. The lowest correlation with WSS was seen in *ANGPT1* with an average R-value of 0.78.

Genes that were correlated with average diameter change were *VEGFA*, *FGF8*, *COL3*, *ET1*, *TGFβ2* and *HHEX*, in descending order. *VEGFA* had the highest average R value of 1.00 while R of 0.89 for *HHEX* was the lowest value.

**Figure 7:**
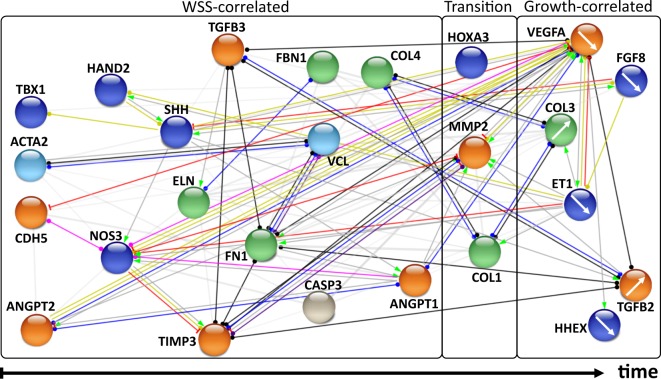
Temporal organization of the gene/protein network during AA morphogenesis. This network was constructed in association with the STRING database showing the selected molecular interactions between genes examined in this study and aligned by time according to the corresponding correlation levels (vertical axis) and peak expression times (horizontal axis). WSS-correlated, both WSS and growth-correlated (transition), and growth-correlated genes were aligned from left to right, respectively. Colours of the nodes represent the biological pathways that genes/proteins involve (orange = angiogenesis, dark blue = cardiovascular development, and remodeling, green = ECM, turquoise = cytoskeleton, grey = apoptosis). Colours of the edges represent the type of interactions (green = activation, red = inhibition, blue = binding, black = reaction, yellow = transcriptional regulation, pink = posttranslational modification, purple = catalysis, grey = unspecified). The directed arrow at the end of edges denotes positive effect, perpendicular line denotes negative effect, and circular shape denotes unspecified effect. White arrows inside the nodes of growth-correlated genes show the trend of gene expression (steady increasing vs. steady decreasing expression).

Peak expression analysis was performed for WSS-correlated genes and the nodes of the network were aligned accordingly. Furthermore, the alignment within each column was arranged in descending order of correlation (Fig. 7). The earliest peak expression in WSS-correlated genes was determined in *TBX1*, *ACTA2*, *CDH5* and *ANGPT2*. The next column included *HAND2* and *NOS3*. The third column included *TGFβ3*, *SHH*, *ELN* and *TIMP3*. *FN1* expression was determined later. *FBN1*, *VCL* and *CASP3* created another column while *COL4* and *ANGPT1* had the latest expression among all. Afterwards, genes with different left and right correlation trends (*HOXA3*, *MMP2* and *COL1*) were aligned to the network. The last group of the network was the growth-correlated genes, which were *VEGFA*, *FGF8*, *COL3*, *ET1*, *TGFβ2* and *HHEX*.

## Discussion

Angiogenesis-related gene expressions other than *TGFβ2* correlated well with the WSS-trend. Only left AA expressions of *ANGPT1* and *TGFβ3* showed inverse of the WSS-trend with a decrease through HH21 following an increase. Other genes in this pathway, including the right AA expressions of *ANGPT1* and *TGFβ3*, followed the regular WSS-trend.

Angiopoietins are important regulators of vascular activation, remodeling and angiogenesis^16^. ANGPT1 is required for embryonic vascular development and ANGPT2 takes a critical role in vascular remodeling by controlling the functioning of other angiogenic proteins^16^. According to the prevailing model of vascular development, VEGFA forms the immature vasculature, and ANGPT1 further remodels and matures the initial vasculature. Moreover, ANGPT1 maintains the stability and quiescence of the vessels while ANGPT2 destabilizes the vessels and promotes sprouting in the presence of VEGFA and regression in the absence of VEGFA^17^. According to our results, left AA expression for *ANGPT2* directly follows the WSS-trend while that of *ANGPT1* inversely follows the WSS-trend. Therefore, *ANGPT2* level is higher in left AA, which implies that the left lateral AA are more unstable than the right. This behaviour can be a cue for the eventual right-sided aortic arch preference in the chick embryos.

ANGPT2 is shear stress-regulated. *ANGPT2* mRNA expression was reported to decrease under dynamic conditions^18^ and low laminar flow upregulated *ANGPT2* mRNA and protein expression while high flow downregulated this gene^19^. However, *ANGPT2* expression was upregulated by the oscillatory shear stress (OSS) and pulsatile shear stress (PSS) *in vitro* and *in vivo*^20,21^. In our experiments, we also observed that *ANGPT2* expression was positively correlated with WSS both for left and right AA. Therefore, it can be concluded that OSS and PSS regulations are partly responsible for the changes in *ANGPT2* expression in AA.

TGFβ signalling plays a significant role in angiogenesis, cardiovascular development and remodeling systems by regulating cellular processes such as cell growth, proliferation, migration, differentiation and even apoptosis^22,23^. In addition, TGFβ signalling enhances the expression of smooth muscle cell proteins and matrix proteins such as fibronectin, collagen and elastin^24^. During embryogenesis, TGFβ2 plays role in the development of AA IV^25^. Studies on AA remodeling showed that *TGFβ2* knockout mice developed hypoplasia and interruptions in AA IV, whereas *TGFβ3* knockout mice had minor changes in curvature of AA without being lethal^26^. In one study, mouse embryonic endothelial cells were exposed to shear stress, and it was seen that *TGFβ3* expression increased with shear stress while *TGFβ2* expression was unchanged. This result also explained the difference in cross-talk between embryonic and adult ECs^27^. We also recorded that *TGFβ3* expression followed the WSS-trend and *TGFβ2* expression did not, which is in correlation with Egorova *et al*.^27^ Furthermore, the constant increase in TGFβ2 might stem from the further maturation of the ECM proteins during later stages.

CDH5 is one of the most critical adhesive molecules spanning the membrane and transferring the information to intracellular compartments in cell-cell junctions^28,29^. It is required for angiogenesis and vascular morphogenesis^30^. As such, it is a part of a mechanosensory complex that senses shear stress^31^. Studies showed that CDH5 levels were increased with shear stress in porcine aortic endothelial cells^32^. Furthermore, shear stress increased CDH5 protein and mRNA expression level in endothelial progenitor cells^33^. Our results also showed the same WSS pattern for *CDH5* expression conforming its role of shear sensing and transducing in chick embryonic AA.

MMPs are important regulators of angiogenesis by degrading the vascular basement membrane and ECM components^34^. On the other hand, TIMPs inhibits the MMP activity, thereby inhibiting angiogenesis. *TIMP3* expression can be induced by shear stress via ETS-1 mediated pathway^35^. Our results also showed that *TIMP3* expression was regulated with WSS. In addition, MMP2 activates latent TGFβ2 during angiogenesis^34^. For both left and right AA, *MMP2* expression significantly increased from HH18 to HH21. This increase might indicate the angiogenic events in the transition from HH18 to HH21. In addition, mechanical stress is known to activate some intracellular pathways which eventually stimulates *MMP2* expression^36^. WSS may also be the reason for the upregulation of *MMP2* through HH21.

Among cardiovascular development and remodeling genes, a WSS-related trend was observed in *NOS3*, *TBX1*, *HAND2* and *SHH* expressions. Gene expressions for *ET1*, *HHEX* and *FGF8* followed growth-trend inversely while *HOXA3* expression had different trends for left and right AA.

NOS3 is expressed in endothelial cells, and has a variety of roles including vasodilation, controlling blood pressure and controlling vascular smooth muscle cell proliferation^37^. It is well established that *NOS3* is a shear stress-responsive gene. *In vitro* studies demonstrate that *NOS3* expression is upregulated with laminar and unidirectional PSS^38^. Indeed, *NOS3* responds to the WSS change relatively fast and reversibly^14^. In addition, *NOS3* expression is seen in the narrow parts of the cardiovascular system where WSS is high. However, *NOS3* expression remains stable in AA of chick embryos^15^. Our results confirm the observations of Groenendijk *et al*. *NOS3* expression in left and right AA followed WSS-trend as expected, but the significant change between different stages was not captured *in vivo*.

Sonic hedgehog signalling pathway is one of the most crucial regulators of cell differentiation and cell proliferation. The most expressed ligand of this pathway, SHH, has significant roles in embryonic development^39^, particularly, cardiovascular and pharyngeal arch development^40^. *SHH* mutant embryos have abnormal AA development and abnormal pathways for NCC migration^40^. TBX1 has roles in AA formation and early remodeling^41^. Heterozygous mutation in *TBX1* gene causes the reduction or complete absence of AA IV while the mutation in both copies disrupts the whole AA system^42,43^. It was found that TBX1 functions as downstream of SHH in pharyngeal arch development^44^. This interaction can be seen in right AA where *TBX1* and *SHH* expression have significantly altered WSS-trend. FGF8 is another molecule necessary for pharyngeal arch and cardiovascular development via controlling the migration of cardiac NCC into the pharyngeal arches. Hypomorphic mutation of *FGF8* causes several AA development defects in mouse embryos including hypoplasia or complete absence of AA IV which eventually results in the aortic arch and subclavian artery defects in adults^45,46^. It was found that *TBX1* and *FGF8* genetically interact in AA development and *TBX1* functions upstream of *FGF8*^47^. Moreover, specific ablation of *TBX1* in pharyngeal endoderm showed that *FGF8* expression was reduced in that region supporting the earlier findings^48^. Our data showed that *TBX1* followed WSS-trend while *FGF8* expression followed inverse growth-trend for both left and right AA. The highest expression of *FGF8* was at HH18 while peak expression of *TBX1* in left AA was at HH21 and right AA was between HH18 and HH21. Therefore, the interaction between *TBX1* and *FGF8* might have a time-lag in the patterning of the aortic arch. In addition, the role of TBX1 in AA IV development is cell non-autonomous^49^, meaning that *TBX1* is not expressed in the components of the vessel itself, but expressed in the pharyngeal endoderm. In our results, we observed the WSS-trend in *TBX1* expression. Thus, endothelial cells lining the AA sense WSS and might induce a signalling cascade eventually activating *TBX1* expression in the pharyngeal endoderm.

ET1 is a peptide having roles in cardiovascular development including AA remodeling and patterning^50^. Homozygous mutation of *ET1* results in the persistence of AA I and II, and hypoplasia of AA IV^51^. It is known that ET1 is shear responsive in such a way that *ET1* mRNA and peptide expression were downregulated with both steady and pulsatile shear stress^52^. Moreover, Groenendijk *et al*. stated that high shear stress areas have lower *ET1* expression^15^. They also semi-quantitatively represented that *ET1* expression in AA increased during the development of chick embryos and assumed that shear stress in AA decreased due to the widening of the lumen. Our results showed that WSS increased in AA with a peak at HH21, and *ET1* expression decreased through HH24 for both left and right AA. This result is in correlation with our previous studies of *ET1* expression in RVA of chick embryos, stating that *ET1* expression decreased with increasing shear stress, and affected by the delay between shear and pressure^14^. Although TBX1 is not related with endothelin signalling in the morphogenesis of AA^53^, FGF8 is related in such a way that *FGF8* is upstream of *ET1* expression in the ectodermal signalling of the first pharyngeal arch^54^. Our results showed that *FGF8* and *ET1* expression followed the same decreasing trend, which might be a clue for their relation in AA. *HAND2* is expressed in the mesenchyme of the branchial arches and neural crest-derived structures in the developing heart^55^. HAND2, required for the survival of NCC in AA, is stimulated by epithelial signalling of ET1, and this signalling cascade regulates the patterning of AA^56^. Mice homozygous mutant for *HAND2* had involute AA and eventually died^57^. We did not observe any relationship between the expressions of *HAND2* and *ET1*, but probably our time scale is late for observing this kind of interaction since the NCC migration is completed by HH18. Our results showed that *HAND2* expression followed WSS-trend for both left and right AA, but the mechanism to induce this expression should be further investigated.

HOXA3 plays a critical role in the development and differentiation of the third pharyngeal arch^58^. In addition, *HOXA3* null mutants have degenerated AA III which eventually results in common carotid artery defects^59^. *HOXA3* expression only showed a significant increase between HH21 and HH24 in left AA. This non-significant change might be explained by the existence of AA III at all the stages. *HHEX* is the early marker of endothelial cells during embryogenesis^60^. It is required for the differentiation of endothelial and hematopoietic lineages^61^. In addition, *in vitro* results showed that it acts as a negative regulator of angiogenesis^62^. Our results showed that its expression decreased through development while the expression patterns of other angiogenic molecules increased which might support the work of Nakagawa *et al*. Furthermore, the decrease in *HHEX* expression makes sense since it is required early in development.

All of the extracellular matrix (*FN1* and *FBN1*), cytoskeleton (*ACTA2* and *VCL*) and apoptosis (*CASP3*)-related genes followed the WSS-trend. FN1 is an extracellular glycoprotein and has major roles in vascular development, growth and migration of endothelial and smooth muscle cells and ECM remodeling^63^. In addition, presence and the stability of fibronectin provide the deposition of several ECM molecules and influence the cell behaviour^64^. It is known that fluid shear stress directly affects FN1 recruitment and organization^65^. Our results showed that also *FN1* expression was directly influenced by WSS in AA. FBN1 is the building blocks of microfibrils which eventually form the elastic fibers of the cell. *FBN1* expression was also directly regulated by WSS, proving the importance of WSS in ECM deposition and vascular remodeling.

ACTA2 is responsible for the contractile function of vascular smooth muscle cells^66^. Previous work has shown that *ACTA2* expression was upregulated in the porcine aortic valve endothelial cells with the exposure to OSS^67^. The same is valid for its expression in AA. VCL is an adapter protein which binds actin filaments to integrins in cell-matrix adhesion and to cadherins in cell-cell adhesions^68^. It was demonstrated that VCL functioned in the alignment of the stress fibers in response to shear stress^69^. Moreover, *VCL* expression was upregulated with prolonged unidirectional pulsatile flow^38^. Recent evidence suggests that vinculin also functions in mechano-regulation of CDH5^70^. Our results confirmed the effect of WSS on *VCL* expression and might show the interaction between VCL and CDH5.

During the normal development of vessels, regression of the existing vessels is important as well as the formation of the new ones. The process is controlled by the balance between proliferation and apoptosis of endothelial cells^71^. CASP3 is the main protein involved in the apoptosis of existing vessels^72^. We thought that CASP3 might have a role in the disappearance of AA II in the transition from HH18 to HH21. We saw a significant increase in *CASP3* expression in the left AA between HH18 and HH21 but did not see the same result in right AA. Since the whole lateral arch tissue was collected and examined, as a limitation, we might not detect the individual changes within each AA vessel.

Hemodynamic changes in the vessels alter the activity of the cells. In particular, the cells lining the vessels sense WSS and react to this biomechanical stress by changing their proliferation, alignment and apoptosis patterns. In the light of these, we hypothesized that genes correlated with WSS should be activated earlier and affect the expression of growth-correlated genes. Thus, the locations of the genes in the network were organized accordingly based on their peak expression times. In this temporal network, it was seen that the growth factors VEGFA and FGF8 were downregulated with the signals coming from WSS-correlated genes. In addition, TGFβ2 was upregulated which might be due to the need for ECM proteins for further development. In general, the structural genes such as collagens were activated relatively later than cardiovascular development and remodeling genes, so those genes likely influence the genes responsible for generating the structural components of the vessels. This time-resolved network approach can help predict the expression patterns of critical genes that are not included in the present investigation. For instance, *PITX2* gene plays a critical role in the left-right asymmetric morphogenesis of AA^8^. In the network, PITX2 is related with FGF8, which has an inhibitory effect (Supplementary Fig. S1). *FGF8* expression decreases during AA development, thus *PITX2* expression is likely to increase and promote asymmetry in AA apparatus. In addition, SMAD family member 2 (SMAD2) plays a critical role in signal transduction initiated by TGFβ to promote the expression of matrix proteins^24^. *TGFβ2* expression increases through HH24 in AA, so *SMAD2* expression is expected to increase since TGFβ2 is shown to activate SMAD2 in the network. The predictive capability of this time-resolved network approach needs to be confirmed through further validation experiments.

The network was constructed as a combination of left and right AA gene expression for convenience; however, most of the mechanosensitive genes investigated in this study had different peak expression time points for left and right AA (Supplementary Table S1). This suggests that the genes with *different* peak expression times at each lateral may be responsible for providing the asymmetry of the aortic arch system.

Aortic arch morphogenesis is a critical process in the global cardiac morphogenesis and errors result in and occur coincident with CHDs. The biological pathways regulating the development and the morphogenesis of AA are regulated with hemodynamic alterations. WSS has a significant effect on the expression of genes regulating different biological pathways in the AA morphogenesis of chick embryos, particularly in angiogenesis, vascular structure and apoptosis-related genes. A comprehensive analysis of fundamental trends is presented with varying levels of symmetry breaking. Peak expression analysis was done and novel time-resolved network maps were constructed to allow the integration of WSS response with the regulatory growth-related gene expression patterns, particularly the growth factors. This novel network approach is the first-step towards predictive computational mechanogenomic models where interaction between the genetic characteristics and the resulting morphology can be estimated for more complex diseased conditions and models.

## Methods

### Chick embryonic aortic arch (AA) collection

Fertilized White Leghorn chicken eggs (*Gallus gallus domesticus* L.) were incubated in an incubator (Kuhl Corp., New Jersey, USA) at constant humidity and temperature (67% RH, 37.5 °C) (Fig. 1a). According to both IACUC in US and EU directives, avian embryos up to embryonic day 7 are not counted as animals and do not require specific ethical regulation. A small window was opened on the egg shell and tissue segment including all AA were collected at embryonic stages HH18, HH21 and HH24 using microsurgical tools^73^. Left-sided and right-sided AA vessels were dissected out separately while removing the excess tissue around the vessels. Following extraction, left and right AA tissues were separately transferred to cold Krebs solution, then to RNAlater (Sigma Aldrich) for further analysis.

### Reverse transcription-quantitative PCR (RT-qPCR) analysis

Total RNA of pooled chick embryonic left and right AA (4–7 embryos for each lateral) at HH18, HH21 and HH24 was extracted using GeneJET RNA purification kit (Thermo Scientific). The purity and concentration of the RNA samples were quantified with NanoDrop 2000c spectrophotometer (Thermo Scientific). cDNA was then transcribed with Maxima First Strand cDNA Synthesis Kit (Thermo Scientific). RT-qPCR was performed using Luminaris Color HiGreen qPCR Master Mix (Thermo Scientific) to measure the expression levels for 20 genes in different biological pathways including angiogenesis (*ANGPT1*, *ANGPT2*, *TGFβ2*, *TGFβ3*, *CDH5*, *MMP2*, *TIMP3*), cardiovascular development and remodeling (*NOS3*, *HHEX*, *TBX1*, *FGF8*, *ET1*, *HOXA3*, *HAND2*, *SHH*), extracellular matrix (*FN1*, *FBN1*), cytoskeleton (*ACTA2*, *VCL*) and apoptosis (*CASP3*). Each sample was loaded on the RT-qPCR plate as triplicates and at least three biological replicates were used for each gene. Forward and reverse primer sequences were designed with Beacon Designer Software (Supplementary Table S2). Thermal conditions for the reaction were: initial denaturation at 95 °C for 10 min followed by a three-step cycle of denaturation at 95 °C for 15 s, annealing at 60 °C for 30 s and extension at 72 °C for 30 s, repeated 40 times. Only *ANGPT1* had a unique annealing temperature of 53°C. Glyceraldehyde 3-phosphate dehydrogenase (*GAPDH)* was chosen as control gene to normalize the relative expression levels of the genes using the 2^−ΔΔC^_T_ method^74^. Moreover, fold changes were represented based on HH18 left AA expressions for all genes because HH18 was the earliest stage and a right AA dominance in gene expression was expected.

### Average WSS and diameter calculation

Data for the cardiac averaged WSS and vessel midpoint diameters were extracted for left and right AA from our previously published studies^2,13^. Average WSS for HH18 and HH24 were calculated by taking the sum of WSS multiplied with its corresponding lumen surface area, and then dividing this sum to the total aortic manifold surface area. For WSS calculations of HH21, the observed probabilities of AA configurations were also taken into consideration^13^. Average diameters for HH18 and HH24 were calculated by taking the arithmetic mean of the AA diameters^2^. For HH21, probabilities of AA configurations were multiplied with the arithmetic mean of AA diameters^13^. Moreover, lumen areas of each individual AA were measured from new IF images with ImageJ for HH18, HH21 and HH24 (n = 8 for each stage) (Fig. 1b). Lumen dimensions were considered circular and diameters of each individual AA were determined to take the average for left and right AA at each stage. This data was used to validate our approach in average diameter calculation from OCT data. Detailed methods for sectioning of AA and IF imaging will be presented in another study. For further analyses, average diameters calculated from OCT data were used.

### Correlation and Peak Expression Time

Gene expression values of left and right AA for each stage were plotted against average WSS values or average diameter changes and classified according to the trend type the corresponding expressions followed (either the WSS-trend or the growth-trend). The coefficient of correlation (R) was assessed (Supplementary Fig. S2). A value of 0.75 was used as a cut-off for correlation with R. Averages of R values for left and right AA were calculated to assess the overall correlation of a gene expression. For genes following the WSS-trend, slopes were calculated from two gene expression points, and a polynomial curve was fitted using QtiPlot^75^. The time points corresponding to the peak points of the fitted expression curve relative to the embryonic timeline were determined as the “peak expression time” values. Deviations more than 2.4 hours from HH21 time point were considered as early or late peak expression (Supplementary Fig. S3).

### Network Construction

Identifying the peak expression time allowed the ordering of molecular pathway maps as a function of the embryonic time. Gene/protein network interactions were determined using Search Tool for the Retrieval of Interacting Genes/Proteins (STRING) Database^76^. When some of the possible interactions (e.g. *NOS3* and *TBX1*) were not available for *Gallus gallus domesticus*, the organism was selected as *Homo sapiens*. Although left and right selection of AA IV for the mature arch of aorta is different between human and chick, our network approach is not affected by this selection since the network covers the interactions as a combination of left and right gene expression for AA. Default parameters of the database were used to obtain the final network structure. All the active interaction sources were selected, and the minimum required interaction score was adjusted as 0.4. Moreover, molecular action mode was selected to see the molecular interactions between genes and proteins.

Nodes were aligned in embryonic time according to their correlation level and peak expression time value. First, WSS-correlated and growth-correlated genes were grouped. The groups were aligned from left to right as WSS-correlated genes, WSS and growth-correlated genes (transition) and growth-correlated genes, respectively. Afterwards, within WSS-correlated genes, nodes were lined up based on their peak expression times. In addition, the nodes were arranged from top to bottom in descending order of correlation. Different colours were used for nodes to show the biological pathways that the genes involve and to highlight the genes whose activities can be predicted.

### Statistical Analysis

GraphPad Prism version 8.0.0 (131) was used to perform statistical analyses. After determining the normal distribution and the homogeneity of the data, two-way analysis of variance (ANOVA) and post-hoc Tukey HSD were performed to assess the significance of gene expression, average WSS and diameter data. The statistical significance level was adjusted to p < 0.05. All data were presented as the mean ± standard deviation.

## Electronic supplementary material

Supplementary Material

## Data Availability

The data that supports the findings of this study are within the paper and its Supplementary Material File.
