# Supplementary material for: Asymmetry in Mechanosensitive Gene Expression during Aortic Arch Morphogenesis
Source: Sci Rep. 2018 Nov 16;8:16948. doi: 10.1038/s41598-018-35127-7 (PMC6240117; doi:10.1038/s41598-018-35127-7)
Supplement: Supplementary file 1 — Supplementary Material [file 41598_2018_35127_MOESM1_ESM.docx]

**Supplementary Material**

**Asymmetry in Mechanosensitive Gene Expression**

**during Aortic Arch Morphogenesis**

Cansu Karakaya, MS^1^, Selda Goktas, PhD^1^, Merve Celik, MS^1^,

William J Kowalski, PhD^2^, Bradley B. Keller, MD^3^, Kerem Pekkan, PhD^1^*

**Supplementary Figure S1. Gene/protein network for prediction.** The use of temporal gene/protein network as a predictive tool is illustrated. Including selected genes (*TIMP2, JUN, FOS, PITX2, BMP2, ICAM1, VCAM1, SMAD2, TGFβ1*) with unknown temporal expression patterns enriches the network constructed in Figure 7. Network was adopted from the STRING database showing the molecular interactions between genes examined in this study and aligned by time according to the corresponding correlation levels (vertical axis) and peak expression times (horizontal axis). Colors of the nodes represent the biological pathways that genes/proteins involve (orange=angiogenesis, dark blue=cardiovascular development and remodeling, green=ECM, turquoise=cytoskeleton, gray=apoptosis, red=predictable genes). Colors of the edges represent the type of interactions (green=activation, red=inhibition, blue=binding, black=reaction, yellow=transcriptional regulation, pink=posttranslational modification, purple=catalysis, gray=unspecified). Directed arrow at the end of edges denotes positive effect, perpendicular line denotes negative effect, and circular shape denotes unspecified effect.


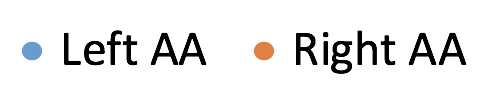


**Supplementary Figure S2. Correlation Analyses.** Gene expression data of left and right AA at HH18, HH21 and HH24 were correlated to corresponding average WSS or diameter and R values were determined **(a)** *FBN1* expression and **(b)** *ANG1* expression were correlated with average WSS while **(b)** *FGF8* expression and **(d)** *HHEX* expression were correlated with average diameter.

**Supplementary Figure S3. Determination of the peak expression time points.** A second order polynomial curve (shown in red color) was fitted to the gene expression level measurement points at HH18, HH21 and HH24 for the genes following the WSS-trend with high correlation. Peak points of the curves (arrow) were determined as the corresponding peak expression time values. Peak points which deviates 2.4 hours from HH21 were considered as early or late peak expression time.

**Supplementary Table S1. Peak expression times of selected genes.** WSS-correlated genes having different and the same peak expression time for left and right AA were tabulated. Genes having different peak expression time at each lateral promotes asymmetry in AA.

| **Different peak expression for left and right AA** | | |
| --- | --- | --- |
| **GENE** | **LEFT** | **RIGHT** |
| ACTA2 | HH18-HH21 | HH21 |
| TBX1 | HH21 | HH18-HH21 |
| FN1 | HH21 | HH21-HH24 |
| HAND2 | HH21 | HH18-HH21 |
| ANGPT2 | HH21 | HH18-HH21 |
| CDH5 | HH21 | HH18-HH21 |
| NOS3 | HH21 | HH18-HH21 |
| VCL | HH21-HH24 | HH21 |
| FBN1 | HH21-HH24 | HH21 |
| CASP3 | HH21-HH24 | HH21 |
| COL1 | - | HH21-HH24 |
| MMP2 | - | HH21-HH24 |
| HOXA3 | - | HH21 |
| **Same peak expression for left and right AA** | | |
| **GENE** | **LEFT** | **RIGHT** |
| ELN | HH21 | HH21 |
| TGFβ3 | HH21 | HH21 |
| SHH | HH21 | HH21 |
| TIMP3 | HH21 | HH21 |
| COL4 | HH21-HH24 | HH21-HH24 |
| ANGPT1 | HH21-HH24 | HH21-HH24 |

**Supplementary Table S2. Forward and reverse primer sequences for the selected genes.** Primer sequences were designed with Beacon designer software.

| **Gene** | **Forward/Reverse** | **Primer Sequence** |
| --- | --- | --- |
| ANGPT1 | F | ATGGAGGAGGATGGACAGTT |
|  | R | CATTTCCCAGCCAGTGTTCA |
| ANGPT2 | F | AGGAGGGTGGTGGTTTGAT |
|  | R | AGTCGTGGCTTTGAGAGAGT |
| ACTA2 | F | GACTGAAGCACCACTGAATCC |
|  | R | GAAGCATACAGGGACAGAACAG |
| CASP3 | F | CAGATGCAAGATCTTTCCCTGG |
|  | R | CCGGTATCTCGGTGGAAGTTC |
| CDH5 | F | GAGCGGTCTCAGGAGGAAT |
|  | R | GAGCACGGACACATCATAGC |
| ET1 | F | CGAGGAGTGCGTGTATTTCTG |
|  | R | CAGCAAGCATCTCTGGCATT |
| FN1 | F | GCAGATCAGACCTCTTGCAC |
|  | R | GTACCCAATAATGGTGGAGGC |
| FBN1 | F | GAAGCAGAGGATGAGCAAGAG |
|  | R | TGAGGGCAGGAAGCAGTT |
| FGF8 | F | AGACCGACACCTTTGGGA |
|  | R | CCTTGCCTTTGCCGTTACT |
| GAPDH | F | GATTCTACACACGGACACTTCA |
|  | R | CTGAGGGAGCTGAGATGATAAC |
| HAND2 | F | CCAGCTACATCGCCTACCT |
|  | R | CCTTCTTCCTCTTCTCCTCCTT |
| HHEX | F | CTACACGCACGCACTGATC |
|  | R | TGGTCTGCTCGTTGGAGAA |
| HOXA3 | F | CTCTTCAAGGCAACGGTTATGG |
|  | R | TTGGCAGAGGGAGGATGAG |
| MMP2 | F | AGGTCGCAATGATGGCAAG |
|  | R | GGCAGCAACCAAGAAGAGAC |
| NOS3 | F | TGGTACATGAGCAGCGAGAT |
|  | R | TCCAGAGGGACGAAGTCTTAC |
| SHH | F | GTCATCGAGGAGCACAGTTG |
|  | R | AGGAGCCGTGAGTACCAAT |
| TBX1 | F | GCTGTGGGACGAGTTCAAC |
|  | R | GGTCCATGCCGAATATCTTCAC |
| TIMP3 | F | CATCGTTATCATCTGGGCTGTG |
|  | R | TCGCTTGGTGTCCTGAGT |
| TGFβ2 | F | CAACTTGGTGAAGGCTGAGT |
|  | R | GTAACGCTGTCCTGGTGATG |
| TGFβ3 | F | TACCTCAGTGGCAGGAATGT |
|  | R | GTATGCCAAGGGCAGTGTATG |
| VCL | F | CGACATCATTGCTGCTGCTA |
|  | R | GTGACTTCATCCGATGCCTTAG |
